# Factors associated with the acceptability of Lopinavir/Ritonavir formulations among children living with HIV/AIDS attending care and treatment clinics in Mbeya and Mwanza, Tanzania

**DOI:** 10.1371/journal.pone.0292424

**Published:** 2024-01-02

**Authors:** Nadiya Alnoor Jiwa, Eunice Ketang’enyi, Kapongola Nganyanyuka, Ruth Mbwanji, Danistan Mwenisongole, Eutropia Masuka, Mary Brown, Mary Charles, Davance Leonard Mwasomola, Thomas Nyangalima, Willyhelmina Olomi, Lilian Komba, Judith Gwimile, Bertha Kasambala, Lumumba Mwita

**Affiliations:** 1 Baylor College of Medicine Children’s Foundation, Mwanza, Tanzania; 2 Mbeya Zonal Referral Hospital (MZRH), Mbeya, Tanzania; 3 National Institute of Medical Research (NIMR)- Mbeya Medical Research Center (MMRC), Mbeya, Tanzania; Kilimanjaro Clinical Research Institute, UNITED REPUBLIC OF TANZANIA

## Abstract

**Introduction:**

Children living with chronic illnesses are offered formulations based on manufacturer and distributor research. The aim of this study is to better understand the perspectives of children and their caregivers in accepting Lopinavir/ritonavir (LPV/r) formulations.

**Methods:**

362 participants were recruited from two pediatric HIV/AIDS clinics in Mbeya and Mwanza, Tanzania, from December 2021 to May 2022. A translated questionnaire was piloted and validated at both clinics, followed by the implementation of a cross-sectional study.

**Results:**

169 participants (47.1%) reported general difficulties in swallowing, regardless of formulation, while 34.3% and 38.5% reported vomiting tablets and syrups, respectively. Statistical significance is shown to support that children can swallow medications if they can eat stiffened porridge (*Ugali*). This correlated with the lower incidence of younger children being able to swallow compared to older children (above six years of age). Children older than six years preferred taking tablets (independent of daily dosage) better than other formulations. Significantly, older children who attend school were associated with high odds of swallowing medicine (AOR = 3.06, 95%CI; 1.32–7.05); however, age was not found to be statistically related to ease of administration for Lopinavir/Ritonavir in this study.

**Conclusions:**

Lopinavir/Ritonavir tablets remain the most accepted formulation among children and adolescents with HIV/AIDS. This study highlights the impact of various factors affecting the acceptability of pediatric formulation, suggesting that children younger than six years, unable to eat *Ugali* and not attending schools may be most vulnerable regarding their ability to accept Lopinavir/Ritonavir formulations. Further studies are needed to assess the acceptability of other medications in chronically ill children.

## Introduction

In 2021, 38.4 million people globally were living with HIV/AIDS. Among children below 14 years of age living with HIV/AIDS, only 52% had access to life-saving anti-retroviral therapy (ART), compared to 76% of adults who could also access ART. This translates into 816,000 children missed from treatment coverage [1]. Sub-Saharan Africa is home to 67% of all people living with HIV [2], as well as 88% of children and adolescents living with HIV (C/ALHIV) globally [3], which implies that the impact of missed antiretroviral therapy would be highest felt in the region. In 2018, Tanzania was estimated to be home to around 150,000 C/ALHIV under 19 years [4].

Research shows that children living with HIV/AIDS in Tanzania struggle to adhere to antiretroviral therapy [5–7] compounded by various socio-economic factors affecting the routine administration of medicines at home.

Crucial aspects related to the ability of a child to take medications on a daily basis include attributes of the medication and the willingness of the child and caregiver to adapt to the offered product determining the acceptability of the said medication [8]. In 2018 the World Health Organization (WHO) defined and unpacked the concept of acceptability related to pediatric formulations and established that the desired clinical balance between stability, absorption, disease characteristics, safety, and cost would ultimately define the outcome of the therapy in children. Acceptability dimensions to support pediatric formulations include palatability; swallowability; dose size, volume, and flexibility; ease of use; dosing frequency, impact on lifestyle; packaging; and transport and storage conditions [9].

This study explores these acceptability dimensions (excluding packaging, transport and storage conditions) from the perspective of caregivers and participants attending specialized pediatric HIV clinics in Mbeya and Mwanza, Tanzania.

## Methods

The study team used an iterative process to develop a questionnaire to assess demographic [10] details, medication administration and the impact of a local staple food *Ugali* [11] on swallowability. The questionnaire utilized a Hedonic scale [12] to assess the extent of liability for each of the four formulations of Lopinavir/Ritonavir (LPV/r) [13, 14] which was formerly recommended as the first line pediatric protease inhibitor antiretroviral therapy for care and treatment of C/ALHIV in Tanzania (replaced by Dolutegravir 10mg tablets in 2021 [15]). These four formulations include: o Lopinavir/Ritonavir 80 mg/ 20 mg per ml syrups o Lopinavir/Ritonavir 40 mg/ 10 mg per sachet granules o Lopinavir/Ritonavir 100 mg / 25 mg pediatric tablets o Lopinavir/Ritonavir 200 mg/ 50 mg adult tablets.

The questionnaire was translated into Kiswahili and piloted with 20 participants at both sites, after which responses were back translated to English and assessed. Kiswahili questionnaire was then changed with synonyms to closely relate to the English version more easily, with minimal distortion to the original intent of the questions.

The sample size estimated that 312 participants would represent 1,648 participants who were on Lopinavir/Ritonavir based treatment regimen for both sites in Mbeya and Mwanza, using Survey Monkey website [16] with 95% confidence interval and 5% margin of error. This study recruited 362 participants using convenience sampling based on their attendance date, of which 128 respondents were from Mbeya and 234 were from Mwanza.

A survey was administered by health care workers. Samples of the formulations were provided during the interviews for recognition and to reduce recall bias. Then administered for participants who were below 18 years of age, having used Lopinavir/Ritonavir based treatment regimen in the past, and attended the clinics between December 2021 to May 2022. Participants who had transferred to another facility or were older than 18 years were excluded from this study. Informed consent was sought from all participants (either child or caregiver who responded to the questionnaire).

## Data processing and analysis

Data entry and cleaning was performed by principal researchers and statistician. Filled forms were coded and stored safely to ensure confidentiality as data was entered by focal persons from both clinics. Data was grouped for 0 to 5.9 years of age and for 6 to 18 years of age, which was further analyzed using STATA version 15 software to describe and relate swallowability, likability, and ease of administration to variables collected from this study, including demographic data. Methods employed for establishing co-relation between variables include Chi-square, univariable and multivariable logical regression analysis. Incomplete forms were not included in the analysis for this study.

### Role of the funding source

This survey was conducted under routine operations of Baylor College of Medicine Children’s Foundation, Tanzania. No specific source of funding was directed for this research.

### Ethics statement

Local IRB approval reference number SZEC-2439/R.A./V.1/124 was received from Mbeya Medical Research and Ethics Review Committee. Written consent was sought from children above twelve (12) years of age, and for younger children, consent was sought from their parent/ caregiver attending clinic with them during their visit day. A copy of the signed page with details of the cross-sectional study and contact details of the principal investigator was provided to all participants in case of future reference. The Signed page was given a code number, similar to the survey forms and stored safely to protect confidentiality of participants. Data entry was done using coded forms only.

## Results

**Fig 1:**
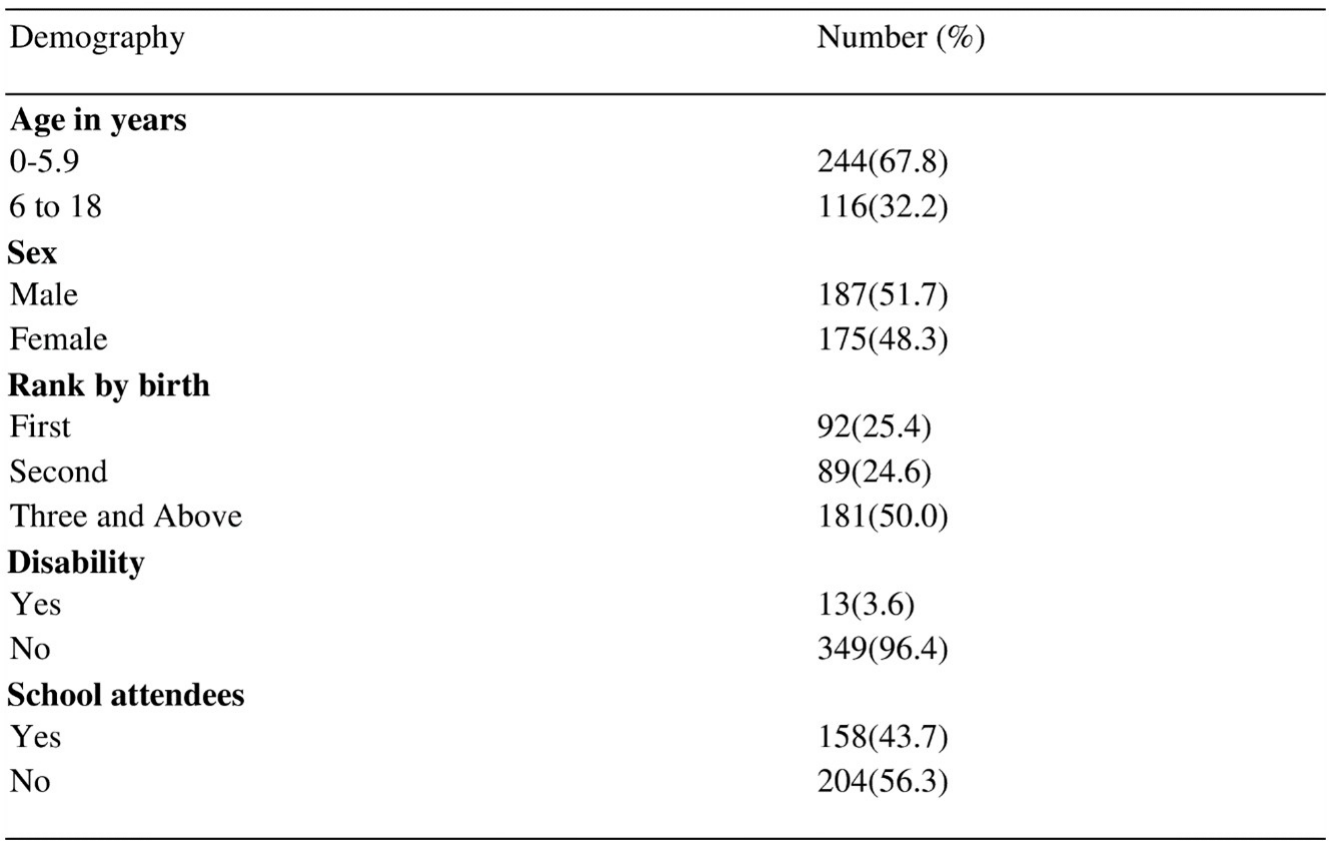
Demographic characteristics of children enrolled in the study.

As shown in Fig 1, most participants taking part in this study were below five years of age (67.8%), 51.7% were male and 96.4% of the respondents had no physical disabilities stated and 56.3% of participants from this study did not attend school.

**Fig 2:**
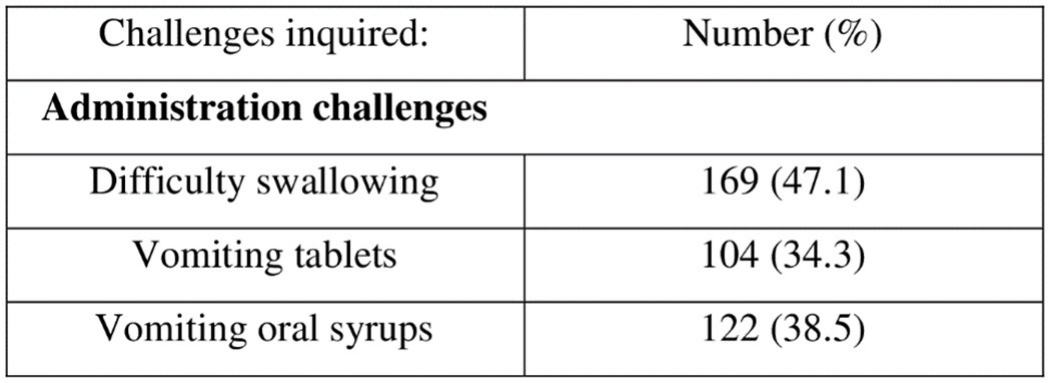
Reported challenges with administering drugs.

Furthermore, Fig 2 shows that nearly half of the respondents (47.1%) reported that their children, at some point or another, while using Lopinavir/Ritonavir based formulations experienced difficulty in swallowing the medications, while 34.3% and 38.5% reported vomiting tablets and syrups at one or more instances during therapy respectively.

**Fig 3:**
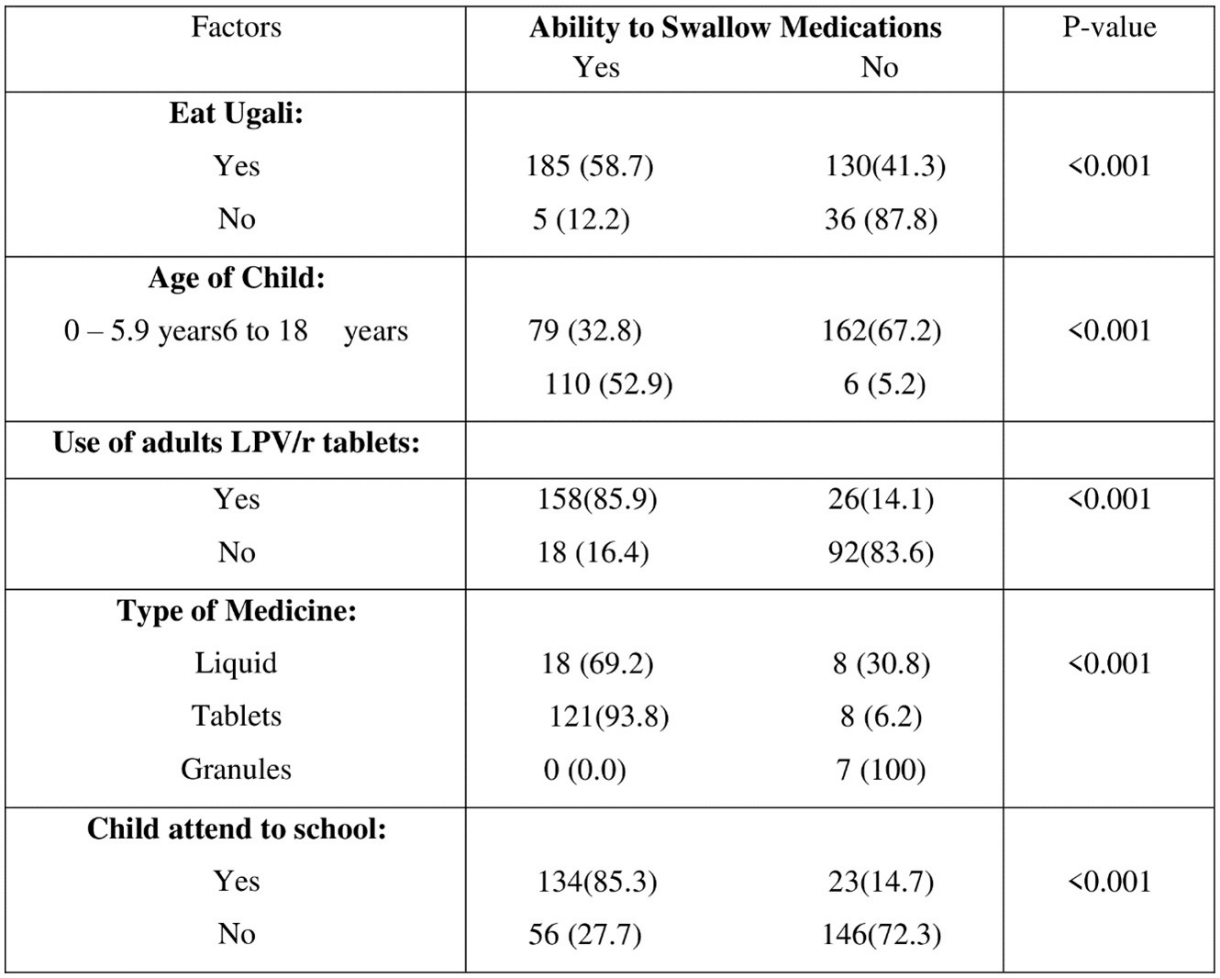
Chi-square test on the relationship between ability of a child to swallow and other factors.

Since the questionnaire was adapted to include culturally friendly food *Ugali* (a common first solid food for Tanzanian children), we related this nutritional practice to the ability of the child to swallow medications based on routine feeding habits at home. *Ugali* is normally chewed or swallowed after dipping in curries and condiments as small hand-kneaded boluses. The results to support a significant relationship between the ability of a child to swallow and other factors are presented in Fig 3.

58.7% participants were able to swallow medications and eat *Ugali* at the same time, which when established through Chi-square test performed for significant difference on the relationship between the ability of child to swallow and other factors, showed that there is a significant association between ability of children to eat *Ugali* and ability of children to swallow.

**Fig 4:**
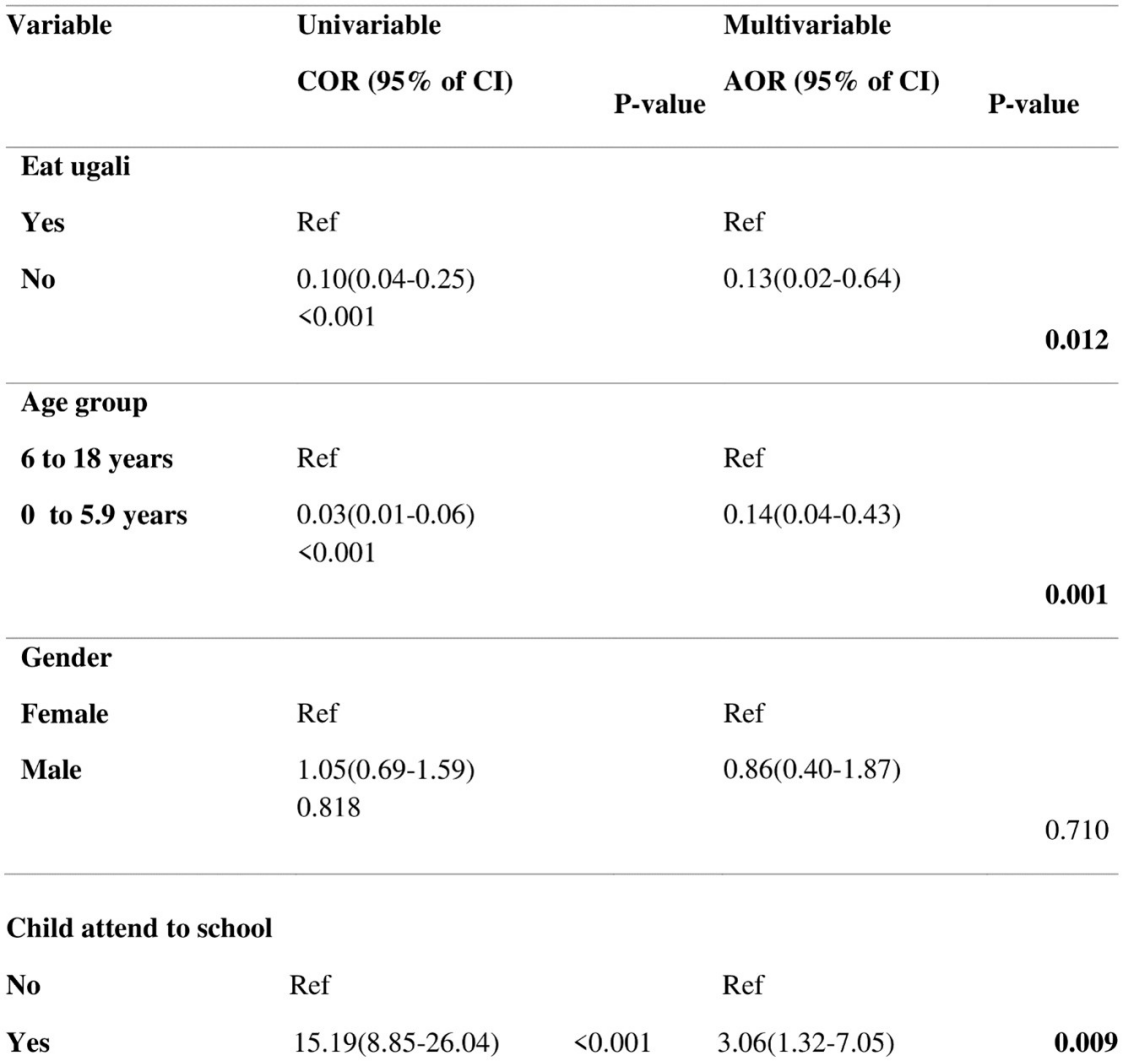
Univariable and multivariable analysis on the factors associated with the child to swallow.

To further assess other factors which would influence child’s ability to swallow medications, apart from habitual/cultural dishes, multivariable logistical regression was also conducted as follows:

Results in Fig 4 for multivariable logistic regression shows that children who do not eat *Ugali* (AOR = 0.13, 95%CI; 0.02–0.64) and are below six years of age (AOR = 0.14, 95%CI; 0.04–0.43) were significantly associated with lower odds of swallowing medicine. This outcome would imply that younger children who may not eat *Ugali* are at disadvantage in their ability to swallow and could need special considerations while being prescribed LPV/r-based regimen in Tanzania.

On the contrary, children aged six years and above, enrolled in schools, associated significantly with ease to swallow medicine (AOR = 3.06, 95%CI; 1.32–7.05), suggesting that children with school enrollment as part of normative social practices influence the ability of a child to swallow medicines.

**Fig 5:**
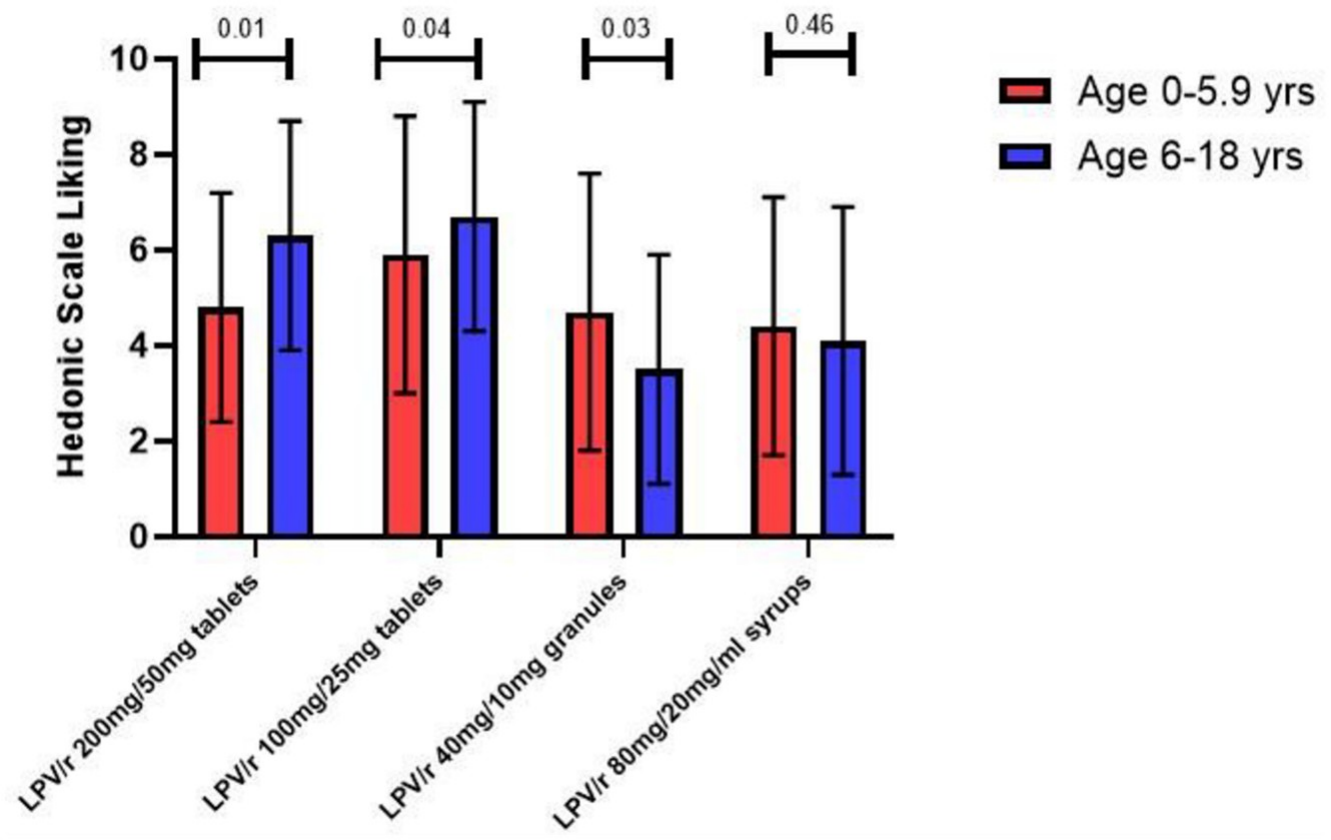
Hedonic scale likability for each of the four LPV/r formulations by age group.

Fig 5 above with Hedonic scale shows that children aged six years and above significantly like Lopinavir/Ritonavir tablets (both adult and pediatric). When compared to younger children, it was found that there was no significant difference for likability syrups or granules formulation.

**Fig 6:**
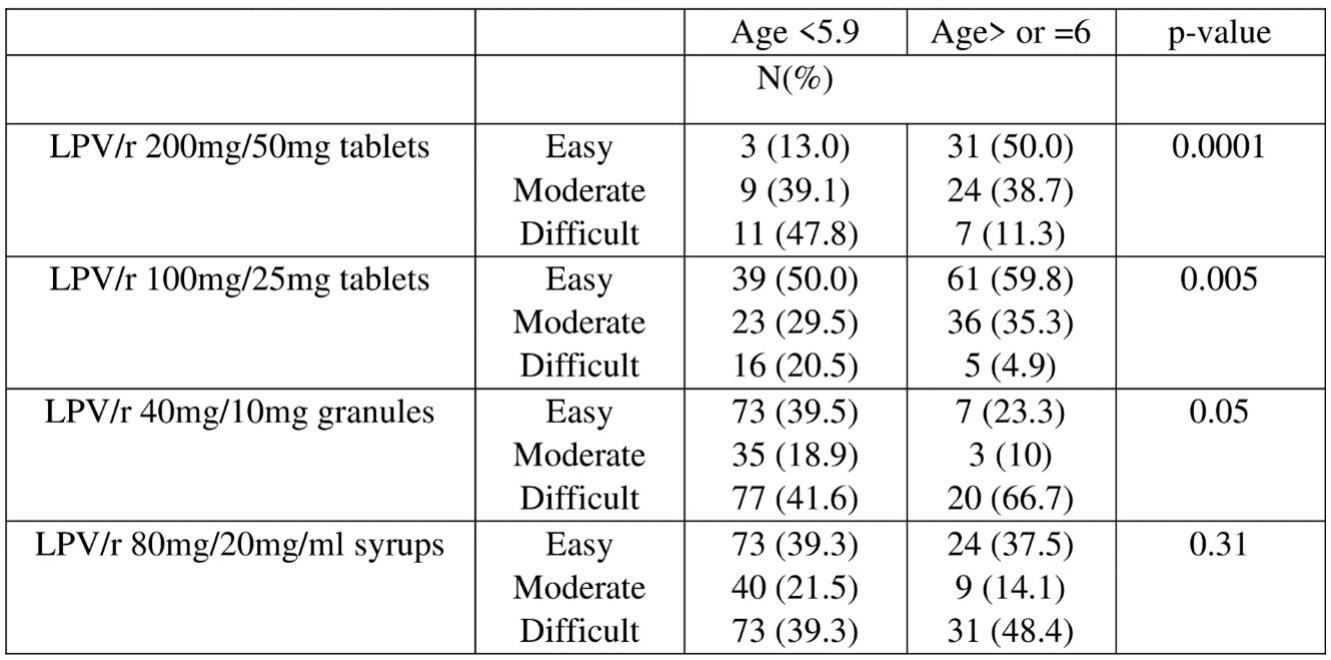
Ease of medication administration by age.

Significant relationship between nature of medication and ease of administration is seen in Fig 6 above, whereby children aged six years and above, find it easier to take both pediatric and adult tablets compared to children below six years, who can take pediatric tablets more easily compared to granules and syrups. Additionally, there is no significant relationship between the ease of administration of granules and syrups and age of the participants.

## Discussion

Acceptability dimensions for pharmaceutical products remain a largely unexplored arena for pediatric formulations in Tanzania. Although standardized research method for acceptability is yet to be assembled [17], palatability and swallowability [18] remain important elements.

Because children often make up a smaller proportion of patients for many diseases and conditions [19], children are often not prioritized by manufacturers for dosage or formulation modifications [20–22] and off-label [23] use of adult formulations for children. This poses a significant risk of increased adverse reactions [24], possible-excipient related poisoning [25], but also a higher risk of therapeutic failure in children compared to adults.

Research shows that both palatability and swallowability of medications remain a challenge in children under four years of age [26] especially for Lopinavir/Ritonavir formulations where caregivers try to crush the pills to facilitate swallowing. Though this consequently reduces bioavailability and efficacy of the LPV/r [27]. Findings supporting the use of tablets in children compared to syrups in this research resonates across various contextual backgrounds [28, 29]. In general, this study outcome can be categorized broadly under two themes:

### What do children like? And, do not like?

Owing to the unpleasant taste of syrups and granules it can be argued that children did not prefer these formulations compared to tablets. However, children under six years of age who used pediatric tablets, liked them nearly as much as older children. In this case, the taste of the formulation may influence choice; compelling children to like pediatric tablets as they could take them with minimum discomfort compared to distasteful syrups and granules.

This finding is triangulated with ease of administration for medication; which also shows that pediatric tablets were easy to administer to all children, whereas syrups and granules were both comparatively difficult.

### How does society (through food and schooling structures) influence the swallowability of medications?

The ability of a child to gulp down stiff porridge (*Ugali*), especially at a younger age, helps to support children to adapt to a lifestyle of daily oral medications. Children under six years of age struggle to swallow pills, though, can do so if supported by society in behavior modifications at home, including enhanced eating habits to include foods such as *Ugali* in routine diet, which implicitly mimics shaping theory adapted for pill swallowing training offered to children in schools in high income countries [30] without any cost implications.

Schooling of older children also affected swallowability, suggesting children’s exposure to social structures creates willingness to propel, compete and survive in life. Kindling passion for life, happiness, and a sense or normality are of primary importance, particularly for a child who is often sick and unable to portray the full potential of their abilities among peers.

## Conclusion

Children are not fragile among HIV/AIDS populations; their developmental milestones and general wellbeing achievement depend on their ability to swallow and accept lifesaving antiretroviral formulations. The Development of formulations catering to the basic needs of children living with HIV/AIDS is necessary to achieve global goals.

Swallowability, age of the child, schooling, and diet on *Ugali* significantly influence children and adolescents’ ability to adapt to pediatric formulations offered in efforts to save their lives. Children and adolescents above six years, attending school and eating *Ugali*, are expected to have better acceptance, resulting in better adherence to therapy, and expected improved viral suppression. Perseverance is needed from the clinical and research community to support younger children in supplying treatment options friendly to their acceptability, especially because taking daily medicines is predicted to be a part of the lifestyle in chronically ill children for a happier and healthier life.

## Supporting information

S1 Data (XLSX)
